# Hydraulic insertions of cochlear implant electrode arrays into the human cadaver cochlea: preliminary findings

**DOI:** 10.1007/s00405-021-06979-z

**Published:** 2021-08-14

**Authors:** M. Geraldine Zuniga, Thomas Lenarz, Thomas S. Rau

**Affiliations:** Department of Otolaryngology and Cluster of Excellence Hearing4all, Hannover Medical School, Carl-Neuberg-Str. 1, 30625 Hannover, Germany

**Keywords:** Insertion tool, Cochlear implant, Atraumatic insertion, Automated insertion, Soft surgery

## Abstract

**Objectives:**

(1) To evaluate the feasibility of a non-invasive, novel, simple insertion tool to perform automated, slow insertions of cochlear implant electrode arrays (EA) into a human cadaver cochlea; (2) to estimate the handling time required by our tool.

**Methods:**

Basic science study conducted in an experimental OR. Two previously anonymized human cadaver heads, three commercially available EAs, and our novel insertion tool were used for the experiments. Our tool operates as a hydraulic actuator that delivers an EA at continuous velocities slower than manually feasible.

*Intervention(s)*: the human cadaver heads were prepared with a round-window approach for CI surgery in a standard fashion. Twelve EA insertion trials using our tool involved: non-invasive fixation of the tool to the head; directing the tool to the round window and EA mounting onto the tool; automated EA insertion at approximately 0.1 mm/s driven by hydraulic actuation.

*Outcome measurement(s)*: handling time of the tool; post-insertion cone-beam CT scans to provide intracochlear evaluation of the EA insertions.

**Results:**

Our insertion tool successfully inserted an EA into the human cadaver cochlea (*n* = 12) while being attached to the human cadaver head in a non-invasive fashion. Median time to set up the tool was 8.8 (7.2–9.4) min.

**Conclusion:**

The first insertions into the human cochlea using our novel, simple insertion tool were successful without the need for invasive fixation. The tool requires < 10 min to set up, which is clinically acceptable. Future assessment of intracochlear trauma is needed to support its safety profile for clinical translation.

**Supplementary Information:**

The online version contains supplementary material available at 10.1007/s00405-021-06979-z.

## Introduction

Cochlear implants (CI) represent the standard of care for severe to profoundly hearing-impaired individuals [1] and have been suggested in the literature as the most successful neuroprosthesis to date [2, 3]. However, high outcome variability in CI recipients can be observed [4, 5]. As a result, further evolution of eligibility criteria, electrode arrays, processor technologies, and surgical technique continues to take place. While it is difficult to isolate the impact from a single variable on audiological outcomes, the previous literature suggests that decreasing the insertion velocity of electrode arrays into the cochlea can significantly lower insertion forces, [6–9] and lower insertion forces decrease the likelihood of intracochlear trauma [10, 11]. Ultimately, atraumatic CI surgery is highly desirable as it is reported to facilitate superior hearing outcomes [12, 13].

However, such a smooth and slow insertion process is limited by manual human kinematics. Kesler et al. observed 0.87 ± 0.32 mm/s is the mean insertion velocity operated by surgeons when they are asked to insert an electrode array (EA) as slow and simultaneously steady as possible [14]. They observed that velocities on the slower end often yielded more pauses and small accelerations during the insertion trial. Automatization of EA insertions promises a solution to reduce intracochlear trauma not only by reducing the abrupt pauses, accelerations, and human tremor, [14–17] but also by performing insertions that can be lowered to velocities slower than manually feasible [6, 9, 18]. Nevertheless, technologies facilitating automated EA insertions are not widely available for clinical use.

Recently, more tangible efforts to implement automated EA insertions in the operating room were reported. Kaufmann and colleagues recently proposed a solution that consists of a powered, compact drive unit operated by a foot pedal [16]. This unit is able to insert EAs at velocities as slow as 0.1 mm/s and allows surgeon’s visualization and manipulation at all times. However, this unit needs to be screwed to the head of the patient to achieve its stable fixation. Another alternative for the automation of EA insertion in the operating is the RobOtol system (Collin Orl, Bagneux, France), which includes a robotic arm that can operate in three different axes, a cart carrying a controller with a human–machine interface [18]. This system has performed successful insertions of cochlear implant EAs in ten patients, using an automated ultra-slow insertion velocity of 0.02 mm/s [18]. However, one may argue that the main drawback of this approach is the need for resources to acquire the equipment and provide additional space in the operating room as well as the potential to significantly increase the intraoperative time for its optimal alignment or setup.

A simpler automated solution for the insertion of EAs could presumably expedite an easy and wide transfer to different clinical scenarios. Hence, our research group developed a novel, simple insertion tool that proposes the use of hydraulic actuation to deliver an EA [19]. The insertion tool consists of a standard single-use syringe that serves as the EA holder and at the same time provides a forward-feed movement in response to an infusion pump. The concept proposes a shift of the ‘complex’ portion of the automation process to a ‘simple’ repurposed standard syringe, which in theory is easily available even in low-resource contexts. In the first description of our tool, resulting forces of an EA inserted into a cochlea model were measured as an indirect means to describe the tool’s ability to perform a continuous insertion at a pre-determined velocity. A testing platform previously designed to measure automated insertion forces [6, 20] was used, with the insertion tool being strongly secured to the platform by means of screws. The resulting force profile revealed a smooth curve of the expected increase in insertion force with increasing insertion depths and without the peaks and accelerations that have been described when performing manually-operated insertions [14–16]. However, it remains to be determined if our novel tool can insert EAs into the cochlea even when it is attached to a human head using a non-invasive approach. In other words, can a surgical retractor with a flexible arm maintain the insertion tool stable or in position despite the tool’s motion required for the EA insertion? Or is the intracochlear advancement of the EA enough to shift the tool, deeming it an unviable approach?

The aims of present study are twofold: (1) to describe the feasibility of our insertion tool to perform EA insertions into human cadaver cochleas at a pre-determined slow insertion velocity while being attached to a human head only using a standard surgical retractor with a flexible arm; (2) to estimate the time required to set up our tool to perform the insertions.

## Methods

**Fig. 1:**
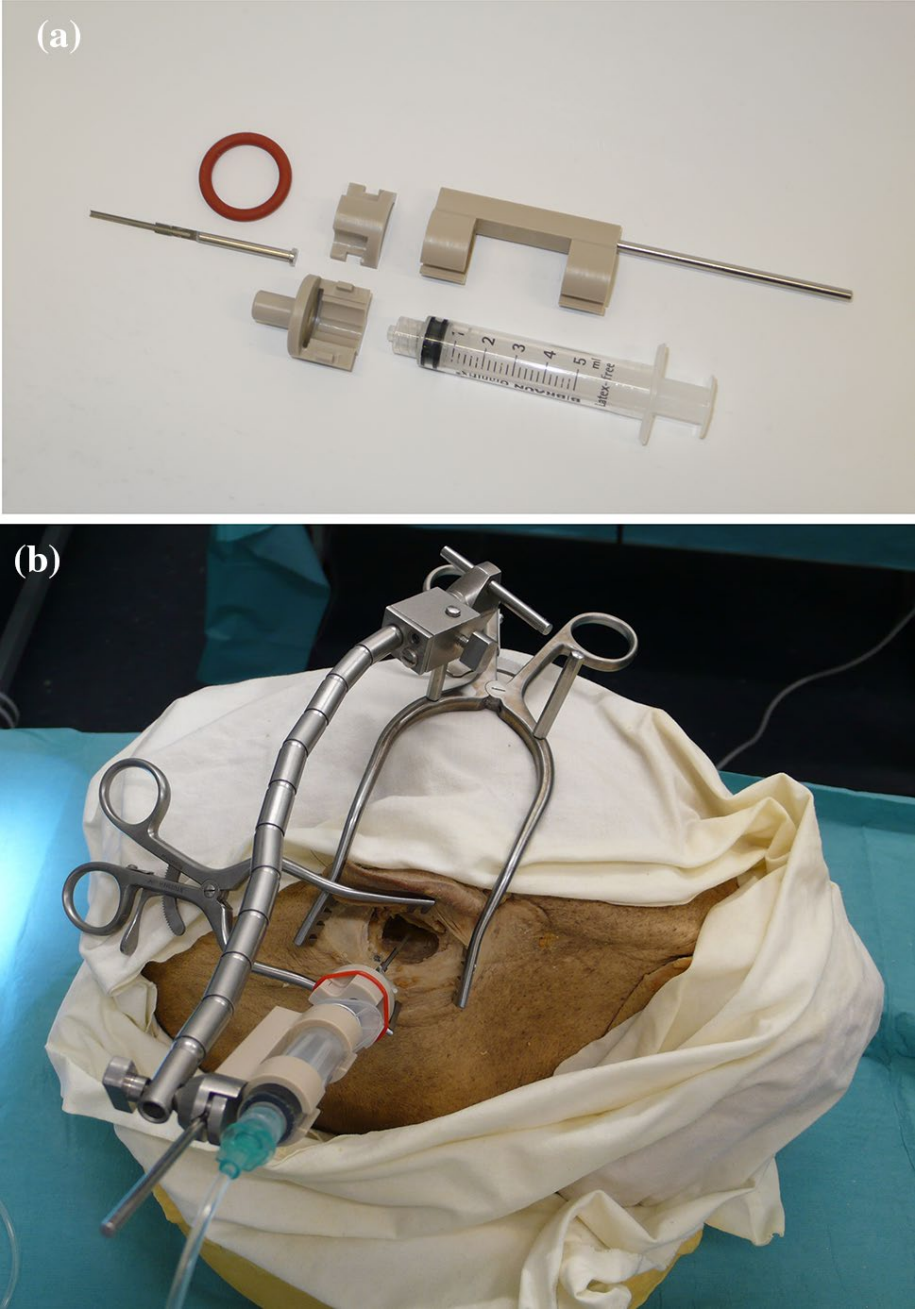
Our novel, simple insertion tool (CHD) before assembly (**a**) and after setup with the retractor holding the insertion tool (**b**)

A novel, simple insertion tool insertion tool and two previously anonymized formalin-fixed human cadaver heads were used to perform EA insertions in three of the four temporal bones. The latter given that the second human head was status post-bilateral transmastoid labyrinthectomy, with a cochlea that was not intact on the right side. Figure 1 illustrates our insertion tool, previously described and named “Cochlea Hydro Drive” (CHD) [19]. Briefly, the CHD repurposes a standard disposable syringe as a hydraulic cylinder that facilitates hydraulic actuation. The tip of the instrument has a shape design that allows the attachment of EAs onto it. The automated, forward motion is then achieved by connecting the insertion tool to a standard medical infusion system. Different infusion rates can then be programmed to drive the insertion tool at different pre-determined velocities, even those below 0.9 mm/s—the slowest limit of manual continuous insertions [14]. The resulting motion from the tool is rather smooth, without abrupt peaks or accelerations [19].

Three commercially available, straight EAs (CLASSIC Series: STANDARD; FLEX Series:FLEX24 and FLEX20 MED-EL, Innsbruck, Australia) were used for the insertion trials. One type of EA featured an intracochlear length of 31.5 mm and was considered the larger EA; the other short EAs had an intracochlear length of 24 mm and 20 mm, respectively. The short EAs are characterized by a thinner apical end (0.5 × 0.3 mm) in comparison to the larger EA (0.5 mm).

All experiments were conducted in a temporal bone laboratory and experimental OR. The present study met review exemption criteria set by our Institutional Ethics Committee.

### CI round window surgical approach

The human cadaver heads were prepared with a round window approach for CI surgery in a standard fashion. Briefly, a roughly 5 cm retroauricular incision was made and the skin flap was raised; an anteriorly based periosteal flap followed. A mastoidectomy and facial nerve recess were drilled to visualize the round window. The overhang of the round window niche was drilled to better visualize the round window membrane, which was fully opened to maximize visualization of the EA insertion.

### Insertion of EAs

An otolaryngologist performed all the EA insertion trials. A standard manual insertion was performed with each EA to establish a control. The surgeon was instructed to perform the insertions as slow and continuously possible, avoiding pauses. The insertion trials using the CHD followed the manual insertions and were conducted as follows: first, the CHD was attached to the specimen with a non-invasive approach using a surgical retractor with a flexible arm (Fig. 1b). The tip of the CHD was directed to the round window. Second, the EA was mounted onto the CHD and its tip was placed right at the round window, the starting position. A standard surgical instrument (e.g., forceps) was used to help position the EA at the starting point. Third, an infusion pump was connected to the CHD using standard clinical tubing including a three-way stopcock valve (Discofix, B. Braun) that allows immediate interruption of the EA insertion if desired. The infusion pump was programmed with an infusion rate of 46 ml/h, which produces a hydraulic actuation of approximately 0.1 mm/s [19]. The beginning of the infusion marked the start of the CHD-insertion, and was directly visualized and video-documented using a surgical microscope (Supplemental Fig. 1). The surgeon verbalized when the EA appeared to have ceased forward movement, indicating the end of the insertion before electrode buckling could occur and a second investigator stopped the infusion using the three-way stopcock valve. This ended the EA insertion. Fourth, following the EA insertion a cone-beam CT scan was obtained to evaluate the intracochlear position of the EA. Of note, the head specimen needed transfer from the surgical table to another table within the scanner, requiring special positioning to fit with the CHD and flexible arm still in place. Finally, the EA and insertion tool were removed from the head specimen and the EA was manually straightened in preparation for the next insertion.

The degrees of EA insertion were determined based on the obtained CT scans using a custom-made DICOM viewer “Comet”, dedicated to cochlear visualization purposes [21]. The middle of the round window is marked manually as the 0 degree reference, and the rotational angle from this point up to the middle of the most apical electrode contact (as the silicon tip was difficult to observe) was calculated [22]. The measuring accuracy of this manual method is within a range of ± 10° [21].

Deviations from the above-mentioned testing protocol, unexpected events, and time spent on setting up the insertion tool were documented.

## Results

**Fig. 2:**
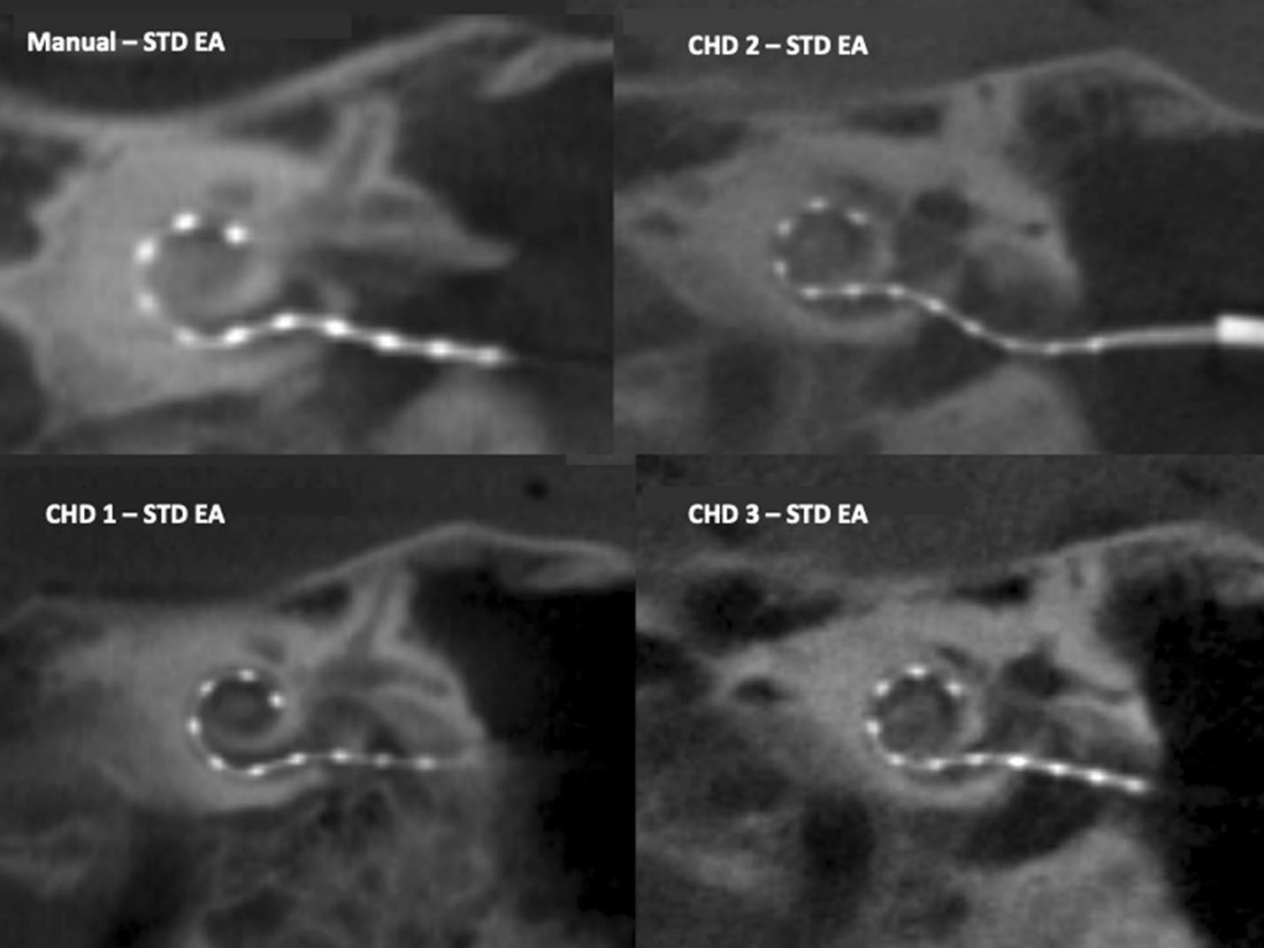
EA insertions performed in temporal bone 1, left side using our insertion tool and a larger EA. A manual EA insertion is provided as reference for this specimen in the left upper corner. See complementary information in Table 1

**Fig. 3:**
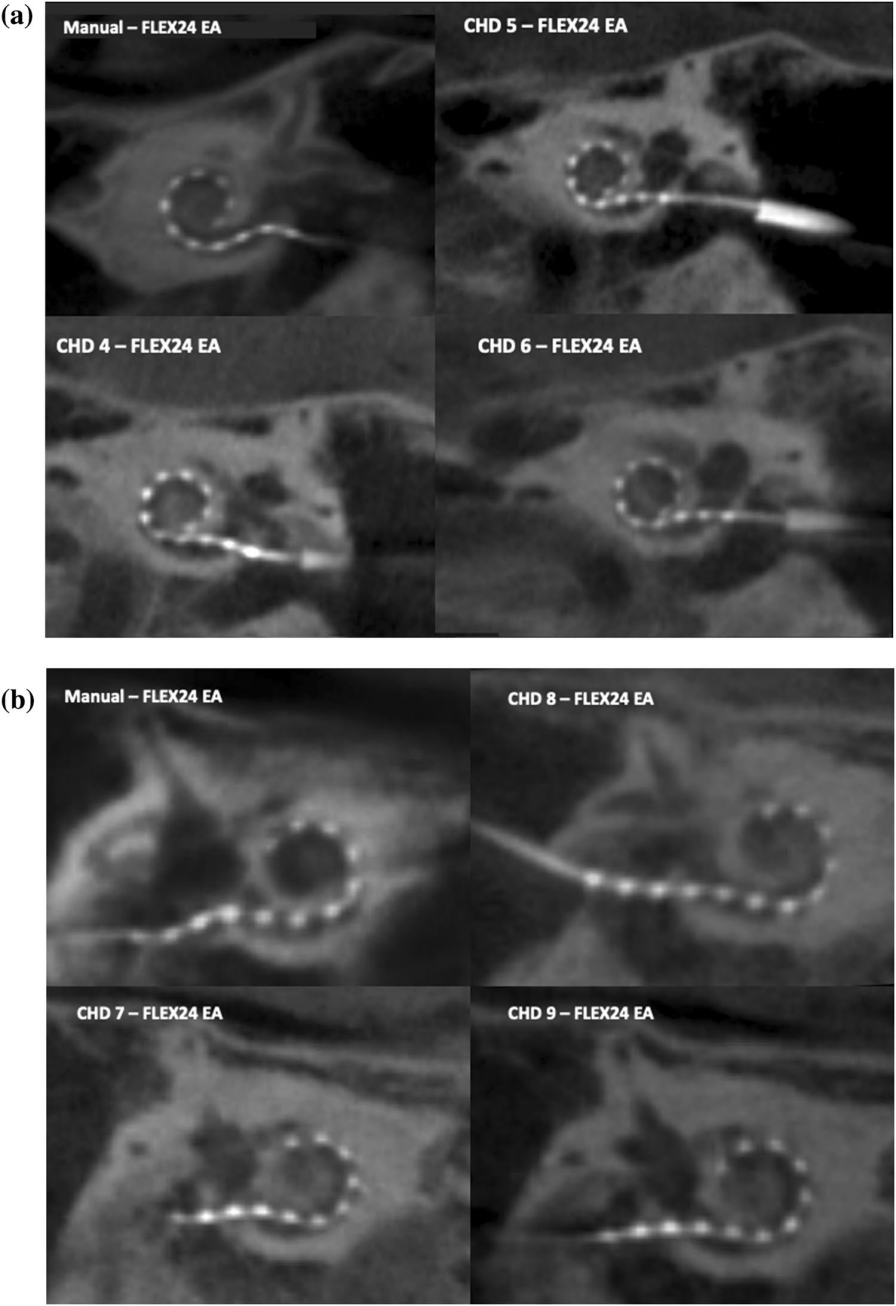
Trials performed with our insertion tool using a 24 mm EA for insertions into the cochlea of temporal bone 1, left side (**a**) and temporal bone 2, right side (**b**). A manual EA insertion reference for this specimen is provided in the left upper corner. See complementary information in Table 1

**Fig. 4:**
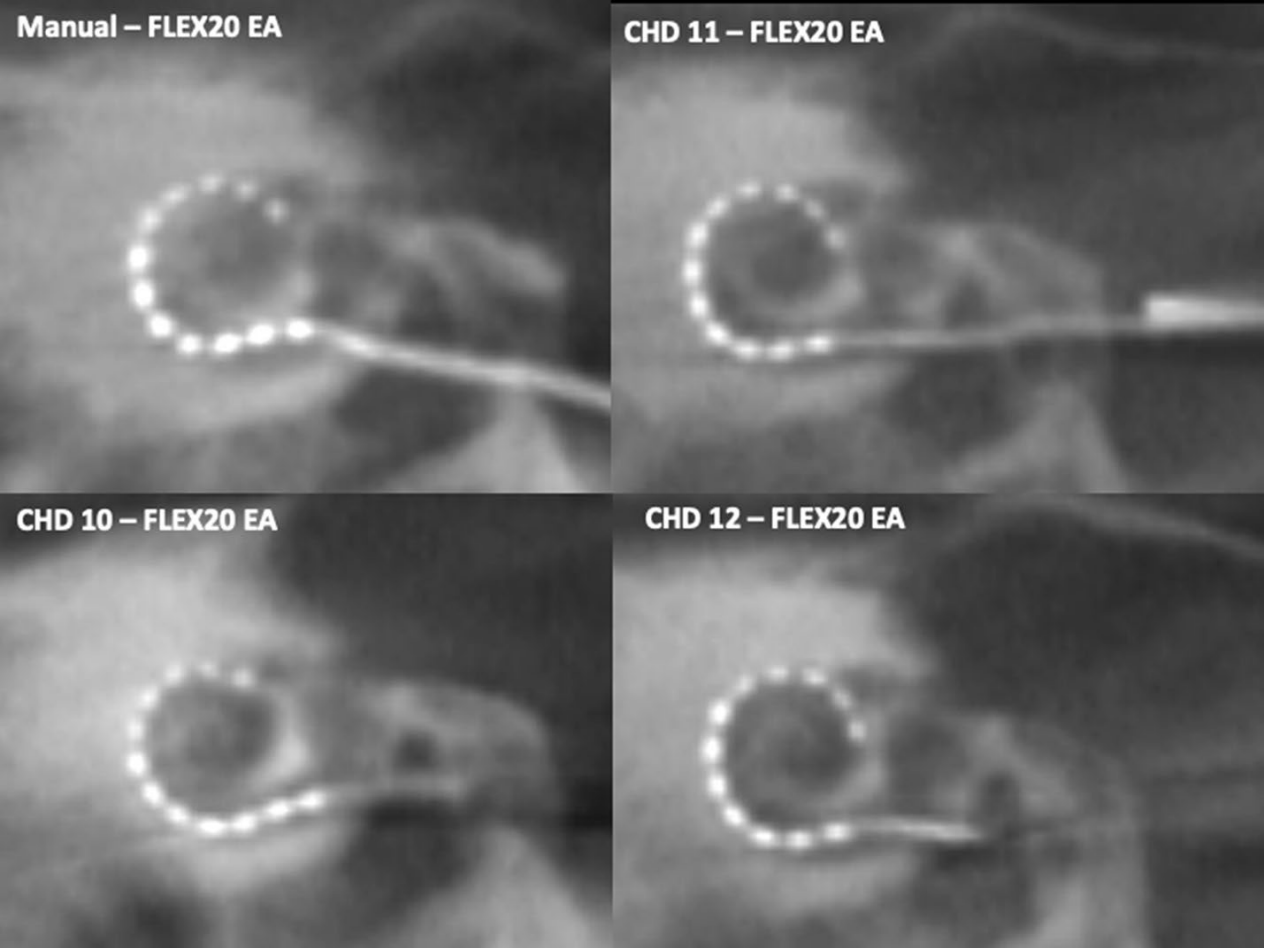
Trials performed with our novel, simple insertion tool using a 20 mm EA for insertions into the cochlea of temporal bone 3, left side. A manual EA insertion reference for this specimen is provided in the left upper corner. See complementary information in Table 1

Twelve insertion trials using the CHD were conducted and intracochlear insertions of the EA were achieved in all cases (Table 1). The manual control using the longer EA on the left side revealed an incomplete insertion of 7 platinum electrode contacts. This limited insertion was anticipated given the previous formalin fixation of the human specimen. Insertion trials # 1–3 tested the same EA with the CHD, and revealed the same number of platinum electrode contacts with intracochlear position (Fig. 2). Decision was made to perform the subsequent insertion trials using the shorter EAs (24 mm and 20 mm). Trials # 4–9 revealed nearly full EA insertions with the 12th electrode at the level of the RW niche, which is comparable to their corresponding manual insertion (Table 1, Fig. 3). The last three trials (#9-12) were performed with the shortest EA (20 mm intracochlear length) and resulted in full insertions both manually and using the CHD (Fig. 4).

**Table 1:** Summary of insertions performed and resulting features

Insertion (#)	Temporal bone (#)	Side	EA length (mm)	Intracochlear platinum contacts	Insertion depth (°)	Insertion time (secs)
Manual-A	1	L	31.5	7	307	90
1	1	L	31.5	7	325	182
2	1	L	31.5	7	299	198
3	1	L	31.5	7	317	204
Manual-B	1	L	24.0	11	405	83
4	1	L	24.0	11	401	210
5	1	L	24.0	11	421	202
6	1	L	24.0	11	413	194
Manual-C	2	R	24.0	11	311	54
7	2	R	24.0	11	292	190
8	2	R	24.0	11	277	178
9	2	R	24.0	11	317	185
Manual-D	3	L	20.0	12	301	71
10	3	L	20.0	12	304	205
11	3	L	20.0	12	312	210
12	3	L	20.0	12	323	195

Manual insertions were instructed as slow and continuous as possible, avoiding pauses and were mainly purposed to test intracochlear patency in these formalin-fixed specimens

Regarding stability of the tool, fixation of the CHD to the head specimen was achieved using a standard surgical retractor with a flexible arm in all 12 trials. Different positions were tried and Fig. 1b depicts a commonly used configuration of the retractor and the insertion tool. Once the tool was attached to the human head and directed to the round window, no accidental movements or position sliding of the instrument were observed for any of the trials. The EA was attached to the CHD, and under visualization using the operative microscope the tip of the EA was positioned at the RW successfully for all 12 trials. The direction and positioning were performed based on surgical visual judgment. The tool remained attached to the human head without need for readjustments for approximately 20 min in each trial (including the time of EA insertion and transfer to CBCT-scanning).

In one insertion trial (#5), the CHD touched the bone covering the facial nerve toward the end of the EA insertion (Supplemental Fig. 2). Of note, once it became apparent that our tool would come in contact with the bone over the facial nerve, we allowed the insertion of the EA to continue as initially programmed. We observed that the hydraulic actuation continued to feed the EA into the cochlea until the insertion was determined completed.

All 12 insertions with our tool were achieved by mere use of the forward-motion provided by the CHD. It was noted that as the EA was inserted into the cochlea, slight shifts in directions of the EA occurred with further advancement of the EA. Post-insertion cone-beam CT scans confirmed the intracochlear position of the EAs (Figs. 2, 3, 4), with a median insertion depth angle of 317 degrees for the larger EA (insertion trials 1–3) and 312 degrees for the smallest EA (insertion trials 9–12). Table 1 summarizes the insertion depths for all 12 trials. No insertion revealed tip fold-over and the EA successfully adopted the expected lateral wall intracochlear position.

Table 2 illustrates the recorded time spent attaching the insertion tool to the human head and overall handling of the CHD to achieve the desired position and direction. The first trial did not include time recordings as we sought to try different ‘accepted’ positions and directions of the insertion tool—i.e., once the surgeon was satisfied with a setup configuration, a new position and/or direction was tested to explore different possibilities for the tool. During trial #2, new setup configurations were still being tested, but these were related to the surgeon getting more familiarized with the insertion tool; thus, the handling times from this and later trials are reported (Table 2). Treating trial #2 as an outlier and considering then the latter trials # 3 thru 6, the median time spend on handling the CHD was 8.8 (7.2–9.4) min (Table 2). Additionally, the recorded videos showed insertions required approximately 3 min with 17 s (3.1–3.4 min) (Table 1).

**Table 2:** Time (seconds) spent in each key step of the insertion using the CHD

Insertion trial (*n*)	2	3	4	5	6	7	8	9	10	11	12
CHD assembly time (s)	87	67	44	75	43	33	36	36	53	39	39
Placing the surgical retractor with flexible arm (s)	16	17	27	13	10	11	36	31	40	48	71
CHD attachment to retractor and direct toward RW (s)	1301	329	118	195	248	132	192	282	268	148	108
Time to mount EA onto CHD (s)	129	78	133	190	170	128	70	76	94	115	75
Fine pre-insertion positioning (s)	383	90	97	0	100	271	201	97	79	0	70
Total CHD-handling ‘additional’ time (s)	1916^a^	581	419	473	571	575	535	522	534	350	363
Minutes	31.93	9.68	6.98	7.88	9.52	9.58	8.91	8.70	8.90	5.83	6.05

^a^Different positions and directions of the insertion tool were still being tested during this trial with the intention to determine a ‘most-suitable’ setup configuration

## Discussion

The present investigation demonstrates that our novel insertion tool achieved ultra-slow, intracochlear EA insertions in all trials (*n* = 12) while being attached to the human head with a non-invasive approach (Table 1, Fig. 1b). The post-insertion CT scans provide evidence of successful position of the EA at the end of each insertion trial and no significant difference to the standard manual insertion (Figs. 2, 3, 4). Hydraulic actuation facilitated the automation of the insertion, revealing smooth, stable insertions that were programmed at a very challenging velocity for the human hand [14]. Furthermore, this very slow actuation is achieved in a *simple* way, taking advantage of an infusion system with a syringe. This is in contrast to other new, elegant but *complex* technologies [16, 18].

The addition of the flexible arm to the surgical retractor to fix the tool in the desired place did not impede appropriate visualization (Supplemental Fig. 1) of the round window and avoids the need for extra invasive procedures. This attachment of the insertion tool to the human head is relevant as it prevents the likelihood of injury during unplanned patient movements (e.g., coughing) that may occur when patients are light on anesthesia. Another advantage of securing our insertion tool using a standard surgical retractor with a flexible arm is that the instrument is already familiar to surgeons (Fig. 1b). Additionally, we observed a quick learning curve for the handling of the CHD, which may be a result of the tool’s simplicity. Only the first two insertion trials required more time due to the surgeon trying different position configurations of our insertion tool before deciding on a setup that seemed to be the most suitable (Fig. 1b). Once these two outliers are excluded, the median time required to position the tool for the insertion was only 8.8 (7.2–9.4) min (Table 2). Based on these advantages, we suggest that our insertion tool has the potential to be widely transferred to multiple clinical scenarios in the future.

Why is it relevant to redirect the automation of EA insertions toward austerity? The WHO estimates the number of individuals in the world living with disabling hearing loss will increase from the current approximate of 489 million to 630 million by 2030, and over 900 million by 2050 [23]. Although not all individuals with disabling hearing loss require a CI, it is logically expected that the number of CI candidates will continue to increase. Therefore, we are urged to optimize CI surgery by developing strategies that not only aim to improve outcomes (e.g., reduce intracochlear trauma) but also to impact a wider range of clinical scenarios. One can presume that disposable syringes and infusion systems are readily available even in the most limited settings and our simple tool should theoretically be able to reach many different clinical environments. In addition, even clinics with a higher infrastructure level may not be able to acquire or adopt complex mechatronic systems for CI surgery.

Furthermore, we suggest that our tool’s setup leads to a clinically acceptable increase in intraoperative time of less than 10 min. Intraoperative time is relevant not only from a medical perspective (e.g., morbidity, time under general anesthesia) but also from a cost and logistic perspective. A previous work evaluating CI operative times at two large CI centers reported average intraoperative times of 2 h and 51 min (95% CI 157–185 min) [24]. Based on these observations, the setup of our tool would be adding slightly less than 10% of surgical time. While OR time costs differ between different institutions and countries, it is safe to suggest that the time and cost added by the CHD would not result in a significant burden. Hence, the current experiments further support that the CHD possesses key features that will allow for a wider clinical transfer than other robotic systems.

In our experiments, once the EA was placed at the round window (starting position for the insertion), no further assistance or re-direction of the EA was performed. This approach may be different based on surgeon’s preference and the entire insertion can be supported by an additional instrument as the EA continues to be inserted by the CHD at a pre-determined ultra-slow insertion velocity. Furthermore, the slight shifts in directions of the EA during its advancement into the cochlea may have occurred due to the turns occurring naturally within the cochlea as the EA advanced, or due to translocations that were not further characterized in the present investigation. These aspects remain to be further studied with the use of specimens that are not previously fixed with formalin.

The present experiments revealed some variability between the trials, manifested as different insertion depths and times. We attribute this variability in large part to our methodology, although some may be due to the use of our tool as well. Regarding different insertion depths, the dimensions of the commercially available EAs play a role, with the larger EA having a larger diameter on its apical end. Also, a slight shift in insertion depth may have occurred as the specimen was transferred and positioned (with the tool still attached) into the scanner. Moreover, the sequential insertion trials may have introduced trauma or bone dust as the same cochlea continued to be used, which may have changed the intracochlear conditions between trials. Furthermore, the use of formalin-fixed specimens made it difficult to target insertions based on merely the dimensions of the cochlea (e.g., cochlear duct length)—as we expected shallower EA insertions. The trials performed with the CHD were concluded based on visual judgment by the surgeon when the EA ceased to advance and before EA buckling could occur. Although the surgeon gained an idea of the feasible insertion depth through the manual insertion, a definite marker or colored reference was not placed on the EA. Therefore, additional preoperative planning that includes accurate individualized determination of EA choice based on a patient’s cochlear dimension will be essential once EA insertions using our tool are performed in patients. Finally, the insertion time variability is explained by the difference in EA insertion length but also by small inaccuracies due to our time recording method—as the magnification required to identify the exact beginning and end of the EA insertion at a speed of 0.1 mm/s would have further limited the view of the surrounding structures in the surgical field.

The major drawback observed during our experiments was related to our fifth insertion trial. In this trial, the final result or intracochlear position was successful (Table 1, Fig. 4), but the CHD touched the bone covering the facial nerve during the insertion (Supplemental Fig. 2). This yielded a valuable lesson that led us to re-think the positioning of the tool as a whole EA + CHD complex. We believe that as the EA had been previously inserted, its shape became inadvertently more curved and resulted in a more posterior position of the tip of the insertion tool as a starting point. In other words, the EA mounted on the CHD was positioned correctly (i.e., the EA tip at the level of the round window), but the angle and direction of the whole EA + CHD complex was suboptimal due to the unnatural shape of the EA that resulted from our previous trials. Hence, ways to improve the alignment of the insertion tool should be further identified. A possible future solution is implementing the use of a more rigid practice dummy EA to position and direct the CHD before the EA that will be inserted is loaded onto the CHD. In addition, a dehiscence of the facial nerve or a very narrow facial recess may be contraindications for the use of the CHD. Finally, one may argue that the success of the insertion in trial #5—despite the suboptimal direction setup—may have resulted from a very smooth insertion facilitated by the programmed ultra-slow insertion velocity of 0.1 mm/s.

Unfortunately, the present study has some limitations that are largely related to our methodology. The experiments were conducted in only three human cadaveric cochleas corresponding to two formalin-fixed human cadaver heads, while only three commercially available EAs of different dimensions were available to perform all insertion trials. The status of the specimens limited further evaluation of intracochlear trauma. However, the use of commercially available EAs was deemed of importance, as the stability and ability of the system to achieve intracochlear insertions was being explored and different EA properties could have misled our conclusions. Furthermore, in these initial insertion trials, we sought to simulate a surgical-like scenario where we would need to also keep the facial recess and facial nerve in the surgical view while the tool is attached in its characteristic non-invasive fashion. Although the formalin-fixed human head specimens limited our ability to explore intracochlear findings, they provided the soft tissue needed to set the retractor with the tool and the same visual field to be expected in the operating room. Further experiments on a larger scale, with fresh human cochleas are required to further validate the performance and reliability of our tool, as well as insertion trauma rates.

## Conclusion

Our novel and simple insertion tool responded to hydraulic actuation to successfully insert an EA into human cadaver cochleas. The minimal invasive fixation of the insertion tool remained stable throughout the insertion process, suggesting that the use of our tool in a clinical scenario is feasible. Furthermore, the setup of our tool requires less than 10 min, which from our point of view is clinically acceptable. Further assessment of intracochlear trauma and insertion trajectory is needed to support its safety profile for clinical translation.

## Supplementary Information

Below is the link to the electronic supplementary material.

Supplementary file1. Surgical view of the EA insertion using the CHD. The EA is held with the tip of the tool (*) (PNG 1849 KB)

Supplementary file2. In insertion trial #5, the insertion tool touched the bone covering the facial nerve (JPG 285 KB)
