# Correction to: Hydraulic insertions of cochlear implant electrode arrays into the human cadaver cochlea: preliminary findings

**DOI:** 10.1007/s00405-021-07052-5

**Published:** 2021-09-03

**Authors:** M. Geraldine Zuniga, Thomas Lenarz, Thomas S. Rau

**Affiliations:** Department of Otolaryngology and Cluster of Excellence Hearing4all, Hannover Medical School, Carl-Neuberg-Str. 1, 30625 Hannover, Germany

## Correction to: European Archives of Oto-Rhino-Laryngology 10.1007/s00405-021-06979-z

In the original version of this article, the author name M. Geraldine Zuniga was incorrectly written as M. Geraldine and the name is corrected in this article.

The original article was updated.

