# Supplementary figures and images for: Hydraulic insertions of cochlear implant electrode arrays into the human cadaver cochlea: preliminary findings
Source: Eur Arch Otorhinolaryngol. 2021 Aug 14;279(6):2827–35. doi: 10.1007/s00405-021-06979-z (PMC9072458; doi:10.1007/s00405-021-06979-z)

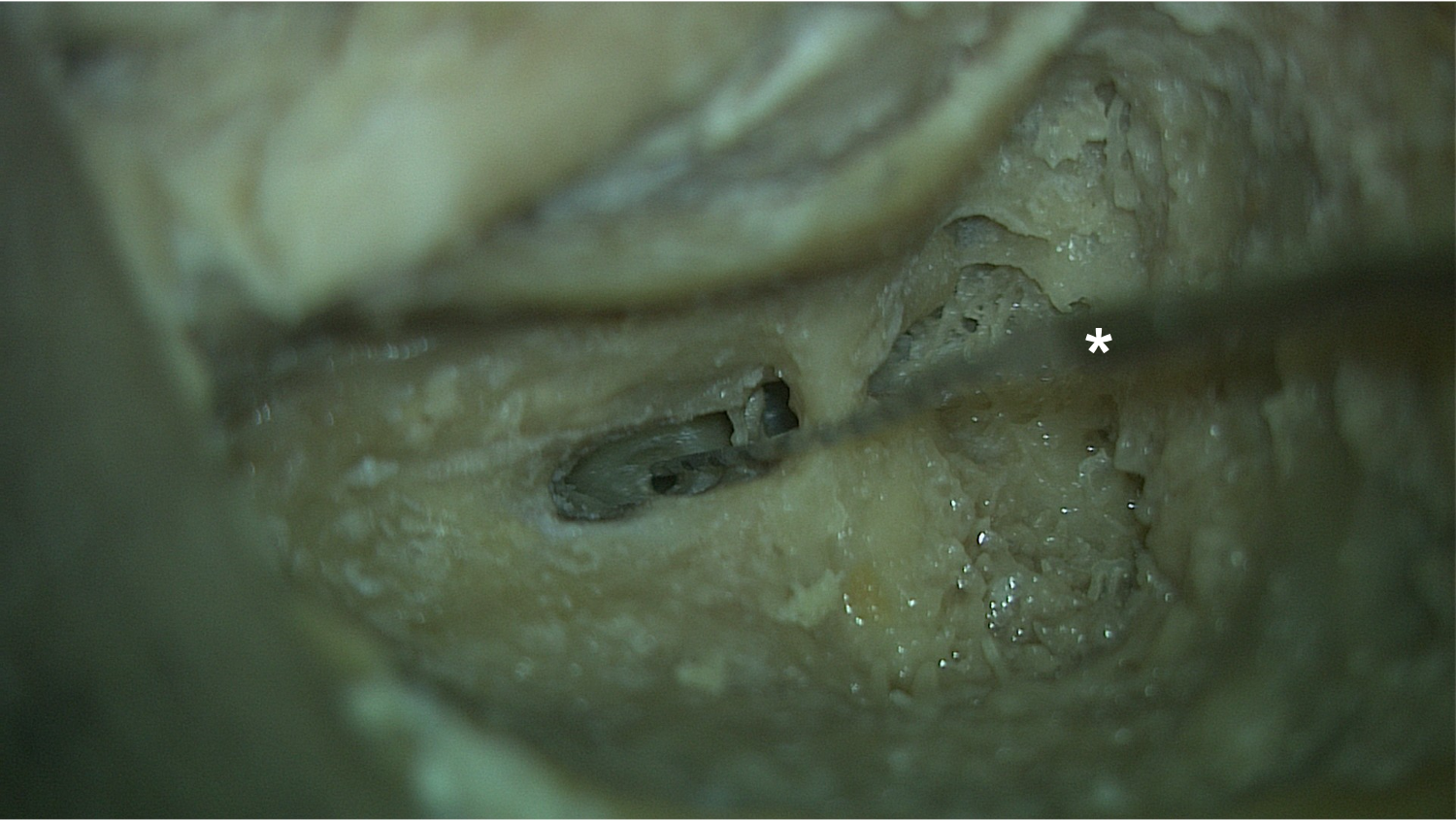

Supplement: Supplementary file 1 — Supplementary file1. Surgical view of the EA insertion using the CHD. The EA is held with the tip of the tool (*) (PNG 1849 KB) [file 405_2021_6979_MOESM1_ESM.png]

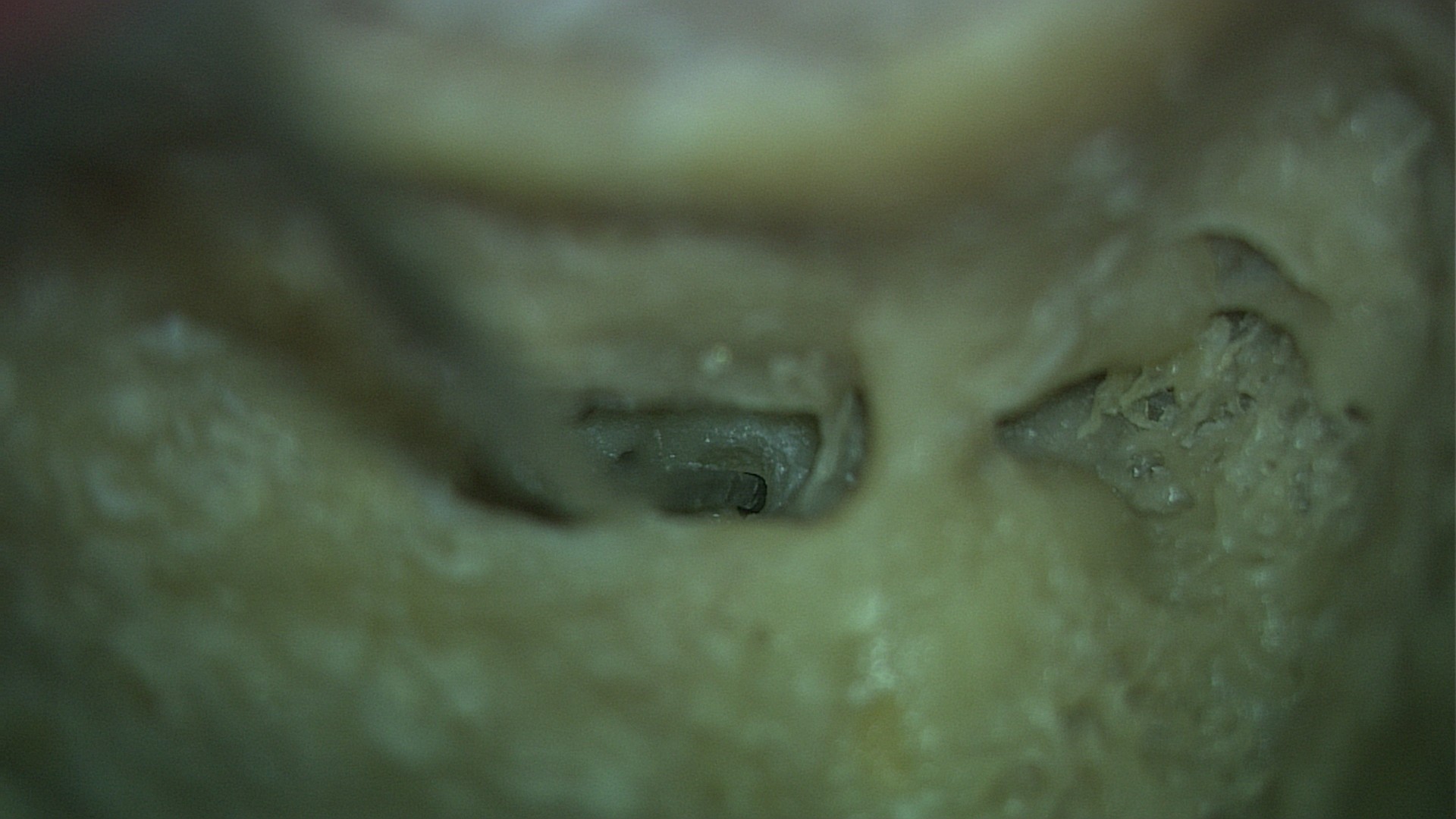

Supplement: Supplementary file 2 — Supplementary file2. In insertion trial #5, the insertion tool touched the bone covering the facial nerve (JPG 285 KB) [file 405_2021_6979_MOESM2_ESM.jpg]
